# Nonalcoholic Fatty Liver Disease and Sarcopenia: Where Do We Stand?

**DOI:** 10.1155/2020/8859719

**Published:** 2020-11-02

**Authors:** Ivana Mikolasevic, Tajana Pavic, Tajana Filipec Kanizaj, Darija Vranesic Bender, Viktor Domislovic, Zeljko Krznaric

**Affiliations:** ^1^Department of Gastroenterology, University Hospital Center Rijeka, Rijeka, Croatia; ^2^Department of Gastroenterology, University Hospital Merkur, Zagreb, Croatia; ^3^School of Medicine, Rijeka, Croatia; ^4^Department of Internal Medicine, Division of Gastroenterology and Hepatology, University Hospital Center “Sestre Milosrdnice”, Zagreb, Croatia; ^5^School of Medicine, Zagreb, Croatia; ^6^University Hospital Centre Zagreb, Department of Internal Medicine, Division of Gastroenterology and Hepatology & Unit of Clinical Nutrition, Zagreb, Croatia; ^7^Department for Gastroenterology and Hepatology, University Hospital Center Zagreb, Zagreb, Croatia

## Abstract

The link between metabolic syndrome (MetS) and sarcopenia has not been extensively studied, but it is evident that they share several common features. Crucial mechanisms involved in sarcopenia-nonalcoholic fatty liver disease (NAFLD) interplay are based on effects of insulin resistance, chronic inflammation, oxidative stress, and crosstalk between organs by secretion of cytokines (hepatokines, adipokines, and myokines). Currently, published studies confirm the association of sarcopenia with the degree of NAFLD defined by liver histology. However, prospective studies that will give us information regarding the causal effect of NAFLD and sarcopenia are still needed. Furthermore, there is a need for a patient-friendly, noninvasive, low-cost method for detection of loss of skeletal muscle mass, strength, and physical performance in the context of NAFLD. Moreover, potential treatment strategies such as physical exercise and nutritional supplementation, that are usually a part of management of sarcopenia, should also be investigated in NAFLD patients, especially given the fact that for now, we do not have a good treatment option for NAFLD. Therefore, future investigations should combine studies on NAFLD and sarcopenia in terms of physical activity and nutritional interventions such as vitamin D supplementation. This review aims to report recent evidence concerning the links between sarcopenia and NAFLD and methods to assess sarcopenia.

## 1. Introduction

During the last few decades, we have witnessed the number of changes due to aging of population, and several growing aging-related health problems need to be addressed by geriatric researchers, including sarcopenia. Sarcopenia was first described at the end of 20^th^ century, and the term is coined using two Greek words: sarx (flesh) and penia (loss) [1]. The first official definition of sarcopenia was given by the European Working Group on Sarcopenia in Older People (EGWGSOP) as a loss of skeletal muscle mass accompanied with low muscle strength and decreased physical performance [2]. More recent guidelines (EWGSOP2) suggest that the first diagnostic criterion for sarcopenia is low muscle strength, which can be easily measured with dynamometry. If the low muscle strength is detected, low muscle quantity or quality confirms sarcopenia [3]. Nowadays, sarcopenia is often considered to be a comorbid disease. Primary sarcopenia is associated with aging (loss of muscle mass and strength), while a secondary sarcopenia develops because of underlying diseases, lack of physical activity, or inadequate nutrition [4]. Sarcopenic patients are at greater risk of a metabolic impairment, prolonged hospital stay, delayed healing, falls, wound infections, and poor surgery outcomes [5].

Prevalence of sarcopenia varies from 6% to 24% (age and gender adjusted), depending on the criteria used to determine muscle mass and strength. The prevalence increases with age, and it can reach >50% after the age of 80 [6]. To some extent, sarcopenia is a physiologic process that starts between the ages of 30 and 40, and it aggravates after the age of 60 when every year 3% of muscle strength is lost [7]. There is no standard or universally efficient therapy for sarcopenia; so, the most important strategies are physical therapy and/or resistance training together with nutrition support. High-protein diet enriched with special anabolic pharmaconutrients (such as b-hydroxy-b-methylbutyrate and leucine) and vitamin D supplementation should be encouraged [8].

Sarcopenia is well defined in the elderly, but it is also often encountered in patients of all ages with acute and chronic muscle-wasting diseases, such as cancer, chronic heart failure, chronic obstructive pulmonary disease, neuromuscular diseases, chronic kidney disease, liver diseases, autoimmune and inflammatory diseases, chronic infection, and polymorbidity [9]. The skeletal muscle is the primary organ of insulin-mediated glucose disposal. Additionally, decreased muscle mass has a crucial role in insulin resistance (IR) and metabolic syndrome (MetS). Thus, it is not surprising that, recently, it was found that sarcopenia is frequently associated with cardiometabolic disorders including MetS, diabetes mellitus (DM), and cardiovascular disease [10]. Also, there is a growing interest in the involvement of skeletal muscle mass in chronic liver disease (CLD), namely, liver cirrhosis, end-stage liver disease (ESLD), and nonalcoholic fatty liver disease (NAFLD) [11].

Sarcopenic obesity is characterized by decreased lean body mass accompanied with excessive adipose tissue accumulation. Obesity aggravates sarcopenia, impairs physical performance, and increases mortality rates [12]. Adipose tissue releases adipokines that regulate lipid metabolism, impact insulin sensitivity, liver fatty infiltration, and fibrogenesis. Also, sarcopenia and sarcopenic obesity are recognized as independent risk factors for the development of NAFLD and liver fibrosis [13].

The link between MetS and sarcopenia has not been extensively studied, but it is evident that there are several common features of both phenomena. Obesity and IR are considered to play the central role in both MetS and sarcopenia [7, 9]. Since NAFLD is regarded as the liver manifestation of MetS, there is an interplay between these two diseases. Patients with MetS are often presenting with loss of muscle mass and the accumulation of intramuscular fat as a result of the complex interplay of inadequate nutritional intake and physical inactivity, insulin resistance, oxidative stress, proinflammatory cytokines, hormonal changes, and mitochondrial dysfunction [7, 9].

Glucose is disposed primarily in the skeletal muscle in an insulin-responsive manner, and the loss of muscle mass may lead to insulin resistance. Furthermore, chronic low-grade inflammation inherent in obesity and central obesity, vitamin D deficiency, physical inactivity, hepatokines, and myokines might play a role in the mechanistic background of sarcopenia and NAFLD [14]. Loss of muscle mass and function induce contractile impairment and plethora of metabolic and endocrine disruptions. Therefore, sarcopenia can affect whole-body metabolism and the immune and inflammatory responses [7]. Sarcopenia could be considered as one of the causative factors for development of NAFLD and should be assessed and tackled as a part of the broad assessment and therapeutic approach to the disease [11]. This review aims to report recent evidence concerning the links between sarcopenia and NAFLD and methods to assess sarcopenia.

## 2. Mechanisms of Interplay between NAFLD and Sarcopenia

**Figure 1:**
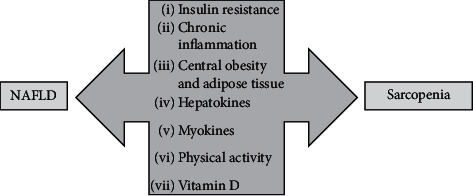
Possible mechanisms of the interaction between NAFLD and sarcopenia. *∗*NAFLD, nonalcoholic fatty liver disease.

According to recent data, sarcopenia is a common complication of liver cirrhosis and is observed in more than half of patients with ESLD [15]. Also, sarcopenic obesity is a common finding in patients with cirrhosis and obesity. Sarcopenia in liver cirrhosis is associated with increased mortality, hyperammonemia and overt hepatic encephalopathy, increased incidence of infections and sepsis, and an increased length of hospital stay after liver transplantation (LT) [16–20]. Moreover, Berzigotti et al., in their two studies, have showed that obesity defined by increased BMI is an important predictor of decompensation of liver cirrhosis in patients with compensated cirrhosis of various etiologies. This effect was independent of some cofounders such as albumin and portal hypertension. According to these studies, liver cirrhosis decompensation occurred in 14% of patients with normal weight, in 31% of overweight, and in 43% of patients with obesity [21, 22]. As it was mentioned, sarcopenia is recognized as one of the risk factors of NAFLD that is the most common cause of CLD and the rapidly rising indication for LT. NAFLD is closely related to MetS, and its individual component, but the main factor, involved in NAFLD pathogenesis is IR [15, 16]. NAFLD is a syndrome that includes a wide spectrum of histopathological alterations ranging from nonalcoholic fatty liver (NAFL) or simple steatosis to nonalcoholic steatohepatitis (NASH) and fibrosis, and finally, cirrhosis and hepatocellular carcinoma (HCC) [15–17]. In recent years, the complex relationship between sarcopenia and NAFLD/NASH has been a focus of research interest [18]. Thus, considerable body of evidence has emerged on the significant interplay between pathophysiological mechanisms of NAFLD and sarcopenia. Given the fact that many of them are shared, it is challenging to decide whether sarcopenia is the cause or the consequence of NAFLD. Crucial mechanisms involved in sarcopenia-NAFLD interplay are based on effects of IR, chronic inflammation, oxidative stress, and crosstalk between organs by secretion of cytokines (hepatokines, adipokines, and myokines) [17–20] (Figure 1).

### 2.1. Insulin Resistance

It is well known that insulin plays a crucial role in glucose metabolism, and that the liver and the skeletal muscle are target organs of insulin. Both the liver and muscle glycogen contribute to the homeostasis of the energy metabolism in the human body. IR is a pathological condition in which cells fail to respond normally to the insulin [17]. IR is a consequence of fat tissue infiltration in the skeletal muscle accompanied by increased circulating free-fatty acid (FFA) from excessive body fat [14]. Furthermore, IR of the skeletal muscle leads to reduction of protein synthesis and increased muscle degradation, which contributes to muscle mass loss. Thus, IR has a pivotal role in sarcopenia development. On the other hand, reduced muscle mass promotes IR [14, 17]. Skeletal muscles by the expression of the insulin-dependent transporter GLUT-4 have a primary role for whole-body glucose homeostasis. In the case of decreased insulin sensitivity, the uptake of glucose is impaired, and insulin stimulated glycogen synthesis [14]. Consequently, there is an increased conversion of glucose to the triacylglycerol in the liver, which leads to development of the fatty liver. This process is responsible for hepatic IR. Moreover, obesity promotes an increased flux of FFA [14]. Fatty liver infiltration is connected more to skeletal IR than liver IR in NAFLD patients, and that observation supports the hypothesis that skeletal muscle IR has the pivotal role in NAFLD development [14, 23]. Therefore, IR is the most important pathophysiological mechanism involved in development of sarcopenia and NAFLD. On the other hand, sarcopenia promotes IR, independent of obesity, because the skeletal muscle is the primary tissue responsible for insulin‐mediated glucose disposal. Furthermore, myosteatosis also promotes IR; thus, the presence of both sarcopenia and obesity are acting together in promoting IR and dysglycemia. The presence of both components, liver injury and sarcopenia, independently or in combination with other confounders, such as obesity, aging, and diabetes mellitus type 2 (T2DM), acts synergistically leading to progression of IR and dysglycemia. IR induces disturbances in function of skeletal muscles, the liver, and adipose tissue [15, 24, 25].

### 2.2. Adipose Tissue

Obesity is a global health problem and an increasing global burden of metabolic, cardiovascular, and malignant morbidity and mortality. A reciprocal interaction among sarcopenia and excess visceral fat aggravates loss of muscle mass [14]. Adipose tissue is the third player in the field of interaction between NAFLD and sarcopenic muscle. Its effects are most pronounced in obesity. The coexistence of sarcopenia and obesity is defined as sarcopenic obesity and recognized as a chronic inflammatory state. Adipose tissue and skeletal muscle inflammation synergistically lead to liver injury and aggravation of sarcopenia. In obesity, adipose tissue inflammation leads to increased secretion of proinflammatory cytokines (e.g., tumor necrosis factor alfa (TNF-*α*) and interleukin 6 (IL-6)) and adipokines (e.g., leptin, adiponectin, resistin, and irisin). TNF-*α* interferes with insulin receptor activity, whereas IL-6 blocks insulin signaling and glucose uptake leading to deterioration of IR. Adding to the complexity of liver-skeletal muscle-adipose tissue axis, it seems that IL-6 and irisin have both proinflammatory effects when acting as adipokines and anti-inflammatory effects as myokine substances [23, 26–30].

Proinflammatory cytokines such as TNF-*α* and IL-6 decrease the adiponectin level. Additionally, myostatin may simultaneously increase adipose tissue mass and decrease the level of adiponectin secretion in adipose tissue. Low level of adiponectin is related with decreased insulin signaling and fatty acid *β*-oxidation in the liver and muscles cells, encouraging an important pathophysiological mechanism in NAFLD and sarcopenia [23, 26–30]. The interplay between adiponectin and myostatin actions within the muscle, liver, and adipose tissue is complex, supporting the vicious circle of perpetuation of all involved mechanisms in damage of target organs.

Increased adipokine leptin secretion in inflammed adipose tissue is associated with decreased energy expenditure, dyslipidaemia, obesity, and IR. In addition, leptin promotes secretion of TNF-*α* and IL-6, strengthening the impact of the inflammatory process in adipose tissue. In NAFLD, it contributes to steatosis and fibrosis, whereas in skeletal muscles, it acts as an anabolic substance. In addition to adipose tissue, leptin could be secreted by skeletal muscles too. Interestingly, when secreted as myokine, leptin acts as a liver protective substance. Unfortunately, the state of sarcopenia limits its autocrine anabolic effects along with remote protective effects on the liver [23, 26–30].

### 2.3. Chronic Low-Grade Inflammation

Inflammation and oxidative stress are shared, and mutually perpetuating pathogenetic mechanisms are involved in IR, sarcopenia, and NAFLD. In addition, obesity, often coexistant with NAFLD, is also recognized as a chronic inflammatory state characterized by increased levels of cytokines and infiltration of adipose tissue with proinflammatory cell types, most notably macrophages. TNF-*α* acts by stimulating reactive oxygen species production and causes oxidative stress and mitochondrial dysfunction. Additionally, it also inactivates the AMP-activated protein kinase pathway, which relates to NAFLD development. Proinflammatory cytokine IL-6 also plays an important role in systemic inflammation and NAFLD/NASH development. Both cytokines have a negative association with the skeletal muscle. Many studies have confirmed the association of high systemic levels of cytokines (e.g., TNF-*α*, high-sensitivity *C*-reactive protein (hs-CRP), and IL-6) with low muscle mass and progressive course of NAFLD [18, 24, 28, 31]. For example, Hong et al. [32] showed in their study that patients who had sarcopenia also had higher levels of hs-CRP in comparison to the patients without sarcopenia. Interestingly, authors had showed that hs-CRP levels had a significant negative correlation with skeletal muscle mass index and liver attenuation index. These data are suggesting that inflammation can be involved in the pathogenesis of sarcopenia and NAFLD [32].

### 2.4. Liver and Hepatokines

Excessive FFA oxidation in NAFLD promotes formation of oxygen free radicals, which cause lipid peroxidation and production of proinflammatory cytokines (e.g., TNF-*α*, transforming growth factor‐*β* (TGF-*β*)). Except direct liver injury and subsequent development and progression of liver fibrosis, several hepatokines (e.g., fetuin *A* and *B*, selenoprotein *P*, fibroblast growth factor 21 (FGF12), leukocyte cell-derived chemotaxin 2 (LECT2), and hepassocin (HPS)) are produced. By its auto-, para-, and endocrine function, these affect IR, protein catabolism, lipid metabolism, and sarcopenia, explaining the possible link between the liver, adipose, and muscle tissues [27, 33].

### 2.5. Skeletal Muscles and Myokines

Skeletal muscles account for about 40–50% of lean body mass. Given the fact that it is the most important tissue responsible for insulin-mediated postprandial glucose disposal, skeletal muscles act as a pivotal factor in glucose and energy homeostasis. Loss of muscle mass leads to the metabolic disturbances, decreased insulin action and signaling-IR, reduced gluconeogenesis, glucose intolerance, pronounced production of triacylglycerol, and exacerbation of proteolysis, which eventually lead to the vicious circle of further aggravation of IR, severity of NAFLD, and muscle consumption [34–36]. Therefore, IR is characterized by disruption of protein metabolism because the mammalian target of the rapamycin pathway remains inactive and cannot inhibit autophagy or lysosomal degradation of proteins and organelles involved in muscle catabolism.

Skeletal muscles secrete myokines and peptides involved in pathophysiological mechanisms of NAFLD. Among them, exercise-induced secretion of IL-6 and irisin has a protective role against NAFLD development in obese patients [37, 38]. Irisin plays a critical role in muscle energy metabolism by increasing energy expenditure due to heat loss and the liver by fatty acid *β*-oxidation [39]. IL-6 within skeletal muscle promotes myogenic differentiation, basal and insulin-stimulated glucose uptake, fatty acid *β*-oxidation, and lipolysis. In the liver, IL-6 acts anti-inflammatory by increasing glucose production and fatty acid *β*-oxidation. Unfortunately, with muscle loss, decreased secretion of both protective myokines can be expected. In inflammation and physical inactivity, skeletal muscles produce a TGF-*β* superfamily member—myostatin. Its autocrine actions inhibit muscle growth and differentiation by activation of proteolytic pathways and inhibition of protein synthesis and regeneration. Myostatin receptors are also present on hepatic stellate cells, inaugurating the link between muscle and liver tissues. It is still unknown whether fatty liver promotes sarcopenia by activation of myostatin production in skeletal muscles or whether sarcopenia promotes liver disease by myostatin-related activation of hepatic stellate cells [15, 18, 40]. Furthermore, we know that obesity is associated with low levels of adiponectin. Myostatin also increase adipose tissue mass, which is connected to the decreased adiponectin secretion [15].

All these explain the direct (independent of insulin effects on adipose tissue) relationship between NAFLD and sarcopenia.

### 2.6. Physical Activity

Physical inactivity decreases muscle mass and interferes with the production profile of myokines and their effects on prevention of further muscle loss and accumulation of intrahepatic fat [27]. Myokine irisin secretion is induced by exercise, possibly explaining negative effects of physical inactivity on liver steatosis. There is also a link between physical activity and the production of hepatokines (e.g., exercise promoted decrease in secretion of hepatic and muscle IR promoter fetuin *A* and increase in secretion of myostatin inhibitor follistatin) [27]. Furthermore, loss of muscle strength and continuation of physical inactivity is a risk factor for more progressive muscle loss, fat accumulation, and aggravation of inflammation, leading to the vicious cycle of repetitive physical inactivity and even more pronounced sarcopenia [27].

### 2.7. Vitamin D

Vitamin D receptor is expressed in various cells including the liver and skeletal muscles. In addition to pancreatic beta cells, vitamin D regulates expression of the insulin receptors in peripheral target tissues too. It is a potent arbitrator in development of IR, MetS, NAFLD, and sarcopenia. In muscles, it plays an important role in myoblast proliferation and differentiation, skeletal muscle growth, and as an attenuator of muscle inflammation. In the NAFLD liver, vitamin D deficiency likely contributes to disease worsening by promotion of inflammation‐mediated pathways and amplification of liver fibrosis [41, 42].

## 3. Diagnosis and Assessment of Sarcopenia in Patients with NAFLD

In both the literature and everyday clinical practice, we can find different tools and criteria to measure muscle mass and define sarcopenia. Traditionally, the term sarcopenia has been used to define loss of muscle mass in the aging population [1]. The European Working Group on Sarcopenia in Older People (EWGSOP) and the Asian Working Group for Sarcopenia (AWGS) recommend using the presence of both low muscle mass and low muscle function (strength or performance) for the diagnosis of sarcopenia [2, 3], thus acknowledging the importance of muscle quality and quantity for clinical outcomes. EWGSOP2, the updated consensus paper on sarcopenia, focuses on low muscle strength as a key characteristic of sarcopenia, given that the negative clinical outcomes are limited to patients with impaired muscle strength and/or function [3]. In a recent study, handgrip strength combined with the model for end-stage liver disease (MELD) score was shown to be the superior predictive model among commonly employed techniques to diagnose sarcopenia in cirrhosis [43].

On the other hand, the North American Working Group on Sarcopenia in Liver Transplantation defines sarcopenia using only muscle mass assessed by CT scan at the L3 level based on assumption that skeletal muscle depletion is the most clinically relevant parameter, least susceptible to various influences that can be objectively measured in clinical practice [44]. It has been shown that muscle mass does not always correlate well with muscle strength or function in the cirrhotic population [45, 46]. Furthermore, when compared with the other modalities, CT scan alone can identify the highest percentage of muscle loss in cirrhosis, which poses a significant risk of over diagnosing sarcopenia in patients without cirrhosis [47, 48]. Unresolved issues in diagnosis of sarcopenia in liver disease with no standardized protocols and clear cutoff points have implications for the accuracy and reproducibility of studies in the field and limit its widespread application in the clinical practice.

### 3.1. Assessment of Muscle Mass

One of the issues in defining sarcopenia lies in different skeletal muscle mass indices that have been suggested for its assessment. When evaluating the adequacy of muscle mass, the absolute level of skeletal muscle mass has been used after adjusting for body size using height squared (SM/ht^2^), weight (SM/wt), or body mass index (SM/BMI). SM/ht^2^ was first suggested by Baumgartner et al. in the New Mexico Elder Health Survey [49]. Defined in this way, sarcopenia was significantly associated with physical disability, but subjects with a greater BMI are less likely to be classified as having sarcopenia [50]. Janssen and coworkers proposed weight-adjusted muscle mass index (SM/wt), which is suggested to be the more appropriate index for obese patients [51]. More recently, Foundation for the National Institutes of Health Sarcopenia Project in 2014 recommended adjusting the appendicular lean mass using body mass index (ASM/BMI) to obtain the parameter that is most strongly and directly correlated with weakness and slowness [52]. Present, there is no gold standard for the assessment of muscle mass in patients with NAFLD.

Although traditional anthropometric measures cannot differentiate fat from muscle, some methods, such as midarm muscle circumference (MAMC), midarm muscular area (MAMA = (MAMC)^2^/4 × 0.314), and triceps skinfold (TSF), are still used in clinical practice because they are safe, readily available, inexpensive, and relatively not affected by fluid retention. In trained hands, these measurements have good intra- and interobserver agreement (intraclass correlation of 0.8 and 0.9 for TSF and MAMC, respectively) [53]. Both MAMC and TSF have demonstrated a good prognostic value for mortality among patients with cirrhosis [54], and low MAMC was found to be an independent predictor of mortality after liver transplant [47] and in a large sample of the general male population [55]. MAMC below the 10th percentile of an age- and sex-matched population is considered for the diagnosis of sarcopenia [47].

It has been shown that ultrasound measures of muscle depths can be used to predict overall skeletal muscle mass, and that appendicular lean body mass data reliably correlate with those derived from DXA scores in older adults [56]. The EuGMS sarcopenia group recently proposed a consensus protocol for using ultrasound in muscle assessment, including measurement of muscle thickness, cross-sectional area, fascicle length, pennation angle, and echogenicity [57]. Japanese authors described a method of estimating the cross-sectional area of the psoas muscle in a healthy population [58]. In patients with cirrhosis, iliopsoas muscle index (IP index, iliopsoas muscle area/height^2^) derived by ultrasound showed a good correlation with CT-based measurements of the muscle loss [59]. In the European population, psoas to height ratio was significantly associated with mortality in a cohort of 75 patients with decompensated cirrhosis [60]. In a recent study, authors proposed a model for the evaluation of sarcopenia using ultrasonic measurement of the thigh muscle thickness and body mass index, which is moderately accurate in comparison to psoas CT/MR measurements, with a receiver operating characteristic area under the curve of 0.78 in men and 0.89 in women [61]. Although ultrasound is an important addition to the diagnostic toolbox of sarcopenia with its noninvasive, easy, portable approach, with reliable and valid data available for older adults, more research is needed to validate prediction equations for those with varying health conditions, including chronic liver disease [62].

Bioimpedance analysis (BIA) is a commonly used method for body composition assessments in both clinical practice and research settings. It is a noninvasive, relatively cheap, and simple technique that can measure the volume of fat and lean body mass by estimating total body water. So, body composition assessment from BIA relies on a calibration equation developed using a reference method such as DXA, CT, or MR. For this reason, it is important to standardize the cutoff values for diagnostic purposes in each population. BIA prediction equation to estimate total body skeletal muscle mass (SM) was generated from the study of Janssen and coworkers who validated BIA against SM obtained from MRI. [63, 64] and adjusted muscle mass by weight (SMI = SM/wt, %). Low SMI was defined as a SMI below one standard deviation of young adult values according to the data from the Third National Health and Nutrition Examination Survey (NHANES III) [63]. In a subsequent study, the same group presented skeletal muscle cutpoints for physical disability risk in older adults where in which skeletal muscle was normalized for height. Severe sarcopenia is defined when SMI is ≤8.5 kg/m^2^ (men) or ≤5.75 kg/m^2^ (women) [63, 64]. These cutoff values are used in the EWGSOP consensus when absolute SM is estimated from BIA [12]. As it was mentioned earlier, data from the NHANES III population study showed that severe hepatic steatosis was associated with a decreased risk of sarcopenia as defined by the height-adjusted SMI (odds ratio (OR) 0.63; 95% confidence interval (CI) 0.46–0.87), but at the same time, it was associated with an increased risk of sarcopenia as defined by the weight-adjusted SMI (OR 1.73; 95% CI 1.31–2.28) [65]. These observations suggest that the definition of sarcopenia may explain the conflicting results regarding the relationship between sarcopenia and NAFLD. In a study from Japanese NAFLD population, there was a higher prevalence of reduced muscle mass using sarcopenia index (ASM/BMI) and the skeletal muscle mass/fat mass ratio (SF) compared to the high adjusted appendicular skeletal mass (SMI). Unlike SMI, sarcopenic index and SF ratio correlated with increasing severity of NAFLD (defined by fibrosis stage and NAFLD activity score (NAS)) [66]. There is a significant impact of adiposity on the validity of BIA and other 2 compartment methods for the assessment of fat free mass. However, the overestimation of fat free mass in obesity can be improved by using a correction factor for subjects with BMI ≥ 30 kg/m^2^ [67].

Dual energy X-ray absorptiometry (DEXA) body composition uses low-dose X-rays to provide a whole-body or regional scan and analyze fat, bone mineral, and lean tissues. The method is precise and reproducible (coefficient of variation 0.5%) [68], but cost and access are an issue in many parts of the world [45]. According to the EWGSOP, it represents the preferred alternative method to CT and MRI in the research setting and clinical practice [3]. DXA specific measures of LM include lean mass index (LMI: total LM/height^2^), appendicular lean mass (ALM: arms LM + legs LM), and appendicular lean mass index adjusted for BMI and height (ALMI: ALM/BMI, ALM/height^2^), and the current EWGSOP recommendations focus on cutoff points usually set at −2 standard deviations compared to the mean reference value (healthy young adults) [3]. DXA-derived LM is higher than skeletal muscle mass measured by CT or MRI because it includes the sum of body water, total body protein, carbohydrates, nonfat lipids, and soft tissue mineral [69]. Although there are conflicting reports on the influence of excess body water on DEXA measurements, the use of ALM has been proposed to minimize confounding by ascites in patients with cirrhosis [47]. To avoid possible further overestimation of LM by lower limb edema, a group from Australia proposed a measurement of upper limb LM, which was most strongly associated with waitlist mortality as compared to other body compartments, with a suggested cutoff for sarcopenia of less than 1.6 kg/m^2^ [43, 45]. In contrast to CT and MR imaging, DEXA cannot measure intramuscular fat, which can account for 5–15% of observed muscle mass in obese people [20].

Skeletal muscle cross-sectional imaging with CT or MR imaging is considered to be a gold‐standard tool, but high cost, limited access to equipment, and concerns about radiation exposure (CT) limit their use for routine clinical practice [12]. Both techniques are highly reproducible and can assess muscle quality and quantity, and the accuracy is not affected by hydration status or fluid overload. With the help of a specific software, CT scan can quantify skeletal muscle index (SMI), which is the muscle area on a CT at the level of the third lumbar vertebra (L3) corrected for height (cm^2^/m^2^) [70]. Patients within the spectrum of NAFLD have no defined SMI cutoffs for sarcopenia, except for those with end-stage liver disease. Additional CT-based measures include psoas muscle diameter and area, which require no specialized computer software. There are some conflicting data on the significance of this parameter; some authors describe its good ability to predict a 1-year posttransplantation mortality [71] or mortality on the liver transplantation waiting list, independently of MELD [72], while others question its representativeness and capacity to identify patients with higher waitlist mortality in cirrhosis [73, 74]. CT has the additional ability to determine muscle radiation attenuation (MRA, expressed in Hounsfield Units), a measure of muscle quality which is inversely related to muscle fat content [75]. It has been shown that diabetes mellitus is associated with a lower muscle mass and a reduced MRA [76, 77]. Furthermore, myosteatosis has a role in decreasing skeletal muscle mass in patients with chronic liver disease [78].

### 3.2. Assessment of Muscle Strength

Handgrip strength (HGS) is the most widely used method for determining muscle strength, with a good correlation with leg strength and most relevant outcomes [2, 3, 12]. HGS is currently recommended by both recent international guidelines (European Association of Study of Liver (EASL); European Society for Clinical Nutrition and Metabolism (ESPEN)) in the assessment of all patients with cirrhosis and liver failure [79, 80]. It is usually performed with a calibrated dynamometer using the nondominant hand and averaged after three successful attempts. Patients with NAFLD have been shown to have higher odds for low muscle strength on HGS measurements irrespective of sociodemographic characteristics, weight, metabolic syndrome, and concurrent illnesses [81]. In patients with cirrhosis, studies have confirmed a correlation between decreased HGS and increasing mortality [82, 83].

### 3.3. Assessment of Physical Performance

A number of tests evaluating the physical performance can be used in the assessment of sarcopenia. Short physical performance battery (SPPB) was initially developed in geriatric population and assessed balance, gait, strength, and endurance by examining an individual's ability to stand with the feet together in side-by-side, semitandem, and tandem positions, time to walk 8 ft, and time to rise from a chair and return to the seated position five times, each scored out of 4 [84]. The SPPB allows for risk stratification and classifies the performance as low (0–6), intermediate (7–9), or higher performance (10–12), with the cutoff point for the diagnosis of sarcopenia in the elderly ≤8. Even though data are lacking in NAFLD, a score <10 increases the odds of mortality by 2.5 in patients with cirrhosis [61]. In 2017, Lai and coworkers developed “Liver Frailty Index (LFI)” for the assessment of muscle strength and function in liver disease. The LFI consists of dominant HGS, time to do 5 chair stands and time holding 3 balance positions (feet side-by-side, semitandem, and tandem), and result in a continuous variable that can then be categorized into frail, prefrail, and robust and assessed longitudinally [85]. The LFI has been shown to be a good predictor of both pre- and postliver transplant morbidity and mortality, independent of the severity of the underlying liver disease [85, 86]. As in the case of previous tests, the LFI has not been validated in patients without cirrhosis.

In Table 1, there are techniques and criteria for assessing muscle mass, muscle strength, and physical performance.

## 4. Clinical Data Linking NAFLD and Sarcopenia

Most data connecting NAFLD and sarcopenia come from studies on Asian population, even though studies on Caucasians are also emerging. Most of the studies are published in the last 6 years. After adjustment for confounding factors, most data confirm direct interaction between NAFLD and sarcopenia (Table 2).

In 2014, Hong et al. [32] analyzed 452 participants. NAFLD was diagnosed by liver attenuation index (LAI), obtained by abdominal computed tomography (CT). Sarcopenia was defined by skeletal muscle mass index (SMI) that was obtained by dual energy X-ray absorptiometry (DXA). SMI had a negative correlation with hs-CRP, triglycerides, HOMA-IR, and with total body fat. Patients who had lower muscle mass had more than five times the higher risk of NAFLD even after adjusting for potential confounding determinants [32]. Additionally, study of Lee et al., on subjects from Korean National Health and Nutrition Examination Surveys, indicate a positive association of NAFLD and sarcopenia regardless of MetS and obesity [35]. Similar data were reported by Kim et al. [89] in 3739 Korean patients in whom NAFLD was defined by fatty liver index (FLI) in the absence of other CLD, but in their study, the association was different with respect to the age group and menopause status. Hashimoto et al. [90] analyzed the relationship of liver steatosis and SMI in 145 Japanese patients with T2DM. NAFLD was defined by trainset elastography (TE) with the controlled attenuation parameter (CAP). In this study, SMI showed a significant negative correlation with liver steatosis defined by CAP values, but only in men participants with T2DM. Interestingly, authors have showed that a 1% increment in SMI was associated with a decreased risk for steatosis by 20% in men with T2DM. Wijarnpreecha et al. [91] in their cross-sectional study investigated data of 11325 US participants. NAFLD was defined by US and sarcopenia with the help of BIA. Authors had reported that sarcopenia was an independent predictor of NAFLD and fibrosis [91]. Interesting data were published by Meng et al. [93] where authors analyzed the association between NAFLD and grip strength (GS), which was measured by an electronic handgrip dynamometer in a large population of 20957 Chinese participants. NAFLD was defined by abdominal US. Authors had reported that GS is negatively associated with NAFLD [93]. In these studies, NAFLD was defined by noninvasive methods; however, still the gold standard for NAFLD diagnosis and grading is liver biopsy. Liver biopsy is especially important in terms of differentiation of nonalcoholic fatty liver or simple steatosis from the necroinflammatory form of NAFLD (i.e., NASH). More convincing data are coming from the study of Koo et al. [33]. In this study, NAFLD was defined by liver biopsy in a large cohort of 309 patients. Authors had clearly showed that the prevalence of sarcopenia was related to the severity of NAFLD. Moreover, those participants with sarcopenia had an increased risk for NASH (OR 2.30; 95% CI 1.08–4.93) and significant fibrosis (OR 2.05; 95% CI 1.01–4.16), respectively. These associations were independent of IR and obesity [33]. Similar data were published in 255 Western patients with NAFLD [94] where NAFLD was also defined by liver histology. All of these studies had cross-sectional design; thus, the causal relationship could not be investigated. In the longitudinal study published two years ago, authors analyzed 10534 participants without baseline NAFLD and 2631 participants with baseline NAFLD [24]. NAFLD was defined by hepatic steatosis index (HIS) and sarcopenia by bioelectrical impedance analysis (BIA) [24]. The follow-up period was 7 years. Authors had found that increases in skeletal muscle mass over time had a beneficial effect in terms of NAFLD development and in terms of the resolution of existing NAFLD [24]. As it is clearly shown, most of studies had shown a significant correlation between the NAFLD and sarcopenia. However, opposite data are coming from two recent studies. Peng et al. [65] analyzed 2551 US patients in whom NAFLD was defined by ultrasound. The definition of sarcopenia included both a low muscle mass and poor function. The skeletal muscle index (SMI) was calculated as the absolute muscle mass (kilograms) divided by height^2^ (meters) or total body mass (kilograms). Authors reported that liver steatosis defined by US was related to a decreased risk of sarcopenia when it is defined by height-adjusted SMI. On the other hand, severe US-defined steatosis of the liver was related to an increased risk of sarcopenia when sarcopenia is defined by the weight-adjusted SMI. Authors conclude that definition of sarcopenia is important when we investigate the relationship among sarcopenia and NAFLD [65]. Additionally, Zhai et al. [95] failed to show the association among NAFLD and sarcopenia. Taking together all these data, the relationship of sarcopenia with visceral obesity and IR seems as an important risk factor for NAFLD, which further accelerates NAFLD progression to more advanced stages of CLD. However, prospective studies are needed that will give us information regarding the causal effect of NAFLD and sarcopenia.

## 5. Further Directions

NAFLD and sarcopenia share many of the determinants involved in their pathogenesis, most importantly, IR and chronic inflammation. Because of the overlap in the pathogenesis of sarcopenia and NAFLD, there are still many open questions. First, overlap in the pathogenesis makes it challenging to determine whether sarcopenia is just a complication of NAFLD or risk factor for NAFLD development and progression to more severe stages such as NASH and fibrosis. Due to the fact that currently published studies clearly confirm the relationship of sarcopenia with the degree of NAFLD defined and by liver histology, there is no doubt that the connection between these two entities exists, some even independent of MetS and IR. However, since most of the studies that investigated the relationship between NAFLD and sarcopenia are cross-sectional, the causality still cannot be drawn with certainty. Thus, further prospective studies that will give us an answer if sarcopenia is a consequence or a risk factor for NAFLD are warranted. Second, if the research proves that sarcopenia is a risk factor, treatment strategies such as physical exercise and nutritional supplementation that are dominantly a part of sarcopenia management should be investigated in the context of NAFLD. In other words, given the fact that for now, we do not have a good treatment option for NAFLD, research should combine studies on NAFLD and sarcopenia in terms of physical activity and nutritional interventions such as supplementation of vitamin D. With this approach, we might see the possible effect of the sarcopenia treatment on NAFLD. This is important not only in the context of sarcopenia and NAFLD but also in the context of NAFLD as a multisystemic disease. Recently, Han et al. [18] had showed that patients with both NAFLD and sarcopenia had a higher risk for atherosclerotic cardiovascular disease (OR = 1.83, *P*=0.014) compared with those without NAFLD and sarcopenia. Thus, studies that will involve also extrahepatic manifestations of NAFLD joined with sarcopenia would be of great interest. Third, the role of myokines is the most attractive in the context of sarcopenia because additional knowledge of their role could provide an effective medication which might treat both NAFLD and sarcopenia. Fourth, myosteatosis can have a greater influence on muscle function than muscle mass itself. Thus, it would be interesting to investigate whether myosteatosis is linked to increased morbidity and mortality in the population of NAFLD patients. Fifth, by definition, sarcopenia includes all three components: loss of skeletal muscle mass, strength, or physical performance. According to current guidelines, muscle function is a main determinant in the evaluation of sarcopenia. However, methods for its assessment are not well investigated in the context of NAFLD, which consequently may lead to a lower detection rate of sarcopenia. Therefore, further investigations on the effect of the low muscle function/performance on development and progression of NAFLD are warranted. Sixth, we need studies that will investigate what is the optimal method for detection of loss of skeletal muscle mass, strength, and physical performance in the context of NAFLD. These methods should also be patient-friendly, noninvasive, uncostly, and available in everyday clinical practice.

## Figures and Tables

**Table 1:** Techniques and criteria for assessing muscle mass, muscle strength, and physical performance.

	Sarcopenia criteria	Assessment technique	Adjustment	Cutoff values	
				Men	Women
Muscle mass	EWGSOP [12], FNIH [87]	DXA	ASM	<20 kg	<15 kg
		DXA	ASM/height^2^	<7.0 kg/m^2^	<5.5 kg/m^2^
		BIA	Predicted skeletal muscle mass equation (SM/height^2^)	<8.87 kg/m^2^	<6.42 kg/m^2^
	AWGS [88]	BIA	ASM/height^2^	<7.0 kg/m^2^	<5.7 kg/m^2^
		DXA	ASM/height^2^	<7.0 kg/m^2^	<5.4 kg/m^2^
	FNIH [87]	DXA	ASM/BMI	<0.789 kg/BMI	<0.512 kg/BMI
	NAWGSLT [44]	CT	SMI	<50 cm^2^/m^2^	<39 cm^2^/m^2^

Muscle strength	EWGSOP [12]	Handgrip strength		<27 kg	<16 kg
	AWGS [88]			<28.0 kg	<18.0 kg
	FNIH [87]			<26 kg	<16 kg

Physical performance	EWGSOP [12], AWGS [88]	Gait speed	4-m course	≤0.8 m/s		
		SPPB		≤8 point score		

∗EWGSOP, The European Working Group on Sarcopenia in Older People; AWGS, The Asian Working Group for Sarcopenia; FNIH, The Foundation for the National Institutes of Health; NAWGSLT, North American Working Group on Sarcopenia in Liver Transplantation; DXA, dual energy X-ray absorptiometry; BIA, bioimpedance analysis; ASM, appendicular skeletal mass; SMI, skeletal muscle index; CT, computerized tomography; SPPB, short physical performance battery.

**Table 2:** Clinical studies linking NAFLD and sarcopenia.

Author and year of publication	Study population	Study design	Method of NAFLD detection	Method of sarcopenia detection	Results
Hong et al. 2014 [32]	452 Korean participants	Cross-sectional	CT	DXA	Patients who had lower muscle mass had more than 5 times higher risk of NAFLD

Lee et al. 2016 [35]	2761 Korean participants	Cross-sectional	NAFLD liver fat score, CNS, HSI. Fibrosis by NFS, FIB-4, and Forns index	DXA	Sarcopenia was related to the significant fibrosis. This association was independent of obesity and insulin resistance.

Kim et al. 2016 [89]	3739 Korea participants	Cross-sectional	FLI	DXA, SMI	Low SMI was associated with FLI (i.e., NAFLD)

Hashimoto et al. 2016 [90]	145 Japanese patients with T2DM	Cross-sectional	TE with CAP	DXA, SMI	SMI had negative correlation with CAP values in men participants with T2DM. A 1% increment in SMI was associated with a decrease risk for steatosis by 20% in men with T2DM.

Wijarnpreecha et al. 2019 [91]	11325 US participants	Cross-sectional	US	BIA	Sarcopenia was an independent predictor of NAFLD and fibrosis

Lee et al. 2019 [92]	4398 Korea participants	Retrospective	US	BIA	An increase in fat mass and a loss of appendicular skeletal mass with aging were associated with incident NAFLD

Meng et al. 2016 [93]	20957 Chinese participants	Cross-sectional	US	Dynamometer	GS is negatively associated with NAFLD

Koo et al. 2017 [33]	309 Korean participants	Cross-sectional	Liver biopsy	BIA	The prevalence of sarcopenia was related to the severity of NAFLD; participants with sarcopenia had increased risk for NASH (OR 2.30; 95% CI 1.08–4.93) and significant fibrosis (OR 2.05; 95% CI 1.01–4.16), respectively

Petta et al. 2017 [94]	255 Italian participants	Cross-sectional	Liver biopsy	BIA	Sarcopenia independently associated with the severity of steatosis and fibrosis on liver histology

Kim et al. 2018 [24]	13165 Korean participants	Prospective	HSI	BIA	Increases in skeletal muscle mass over time had a beneficial effect in terms of NAFLD development and in terms of the resolution of existing NAFLD

Peng et al. 2019 [65]	2551 US participants	Cross-sectional	US	SMI—calculated as the absolute muscle mass (kg) divided by height^2^ (meters) or total body mass (kg)	Steatosis defined by US was related to a decreased risk of sarcopenia when it is defined by height-adjusted SMI. Severe US defined steatosis was related to an increased risk of sarcopenia when sarcopenia is defined by the weight-adjusted SMI

*∗*NAFLD, nonalcoholic fatty liver disease; CT, computerized tomography; FLI, fatty liver index; DXA, dual energy X-ray absorptiometry; CNS, comprehensive NAFLD score; NFS, NAFLD fibrosis score; HIS, hepatic steatosis index; SMI, skeletal muscle index; TE, transient elastography; CAP, controlled attenuation parameter; US, ultrasound; BIA, bioimpedance analysis; GS, grip strength.

## Data Availability

The data used to support this study are included within this article.
